# Molybdenum Dopped Copper Ferrites as Active Catalysts for Alcohols Oxidative Coupling

**DOI:** 10.3390/ma12111871

**Published:** 2019-06-10

**Authors:** Gheorghiţa Mitran, Shaojiang Chen, Dong-Kyun Seo

**Affiliations:** 1 Laboratory of Chemical Technology and Catalysis, Department of Organic Chemistry, Biochemistry & Catalysis, Faculty of Chemistry, University of Bucharest, 4-12, Blv. Regina Elisabeta, 030018 Bucharest, Romania; 2 School of Molecular Sciences, Arizona State University, Tempe, AZ 85287-1604, USA; schen178@asu.edu

**Keywords:** oxidative coupling, hydroxyacetone, molybdenum–copper ferrites, methyl acetate, ethyl acetate

## Abstract

Copper ferrites dopped with molybdenum were studied in an oxidative coupling reaction between methanol and ethanol in the gas phase. The catalysts have been characterized by X-ray diffraction, where the presence of ferrite, magnetite, and tenorite phases was observed; scanning electron microscopy; UV-Vis spectroscopy; and Fourier-transform infrared spectroscopy, which highlighted the presence of octahedral coordination of isolated molybdena species. The catalyst with the highest activity in this reaction and with the highest selectivity to hydroxyacetone is the one that presents Lewis sites with weak acidity. The methyl and ethyl acetate selectivities are directly proportional to the Cu/Fe ratio. It has been observed that the presence of reduced copper sites is responsible for the selectivity in esters, while the presence of reduced iron and molybdenum sites is responsible for the acetol production.

## 1. Introduction 

Catalytic transformation of methanol and ethanol in products with higher added value has become more and more interesting due to their renewable characteristics. The condensation in the gas phase is a complex process which involves alcohol dehydrogenation (or oxidation) to aldehyde, aldol condensation, coupling reactions [1]. The products from methanol and ethanol reactions can be classified into non-coupling products (do not form C–C bonds) such as formaldehyde, acetaldehyde, ethers, methyl, and ethyl acetate and coupling products (C–C bonds are formed), e.g., C_3_, C_4_ alcohols. A mixture of methanol and ethanol was used in the “Guebert” reaction in order to obtain superior alcohols (1-propanol, 1-butanol) [2].

The acyloin condensation is an interesting method which involves C–C bond formation and consists of an intermolecular condensation of two molecules of aldehydes; the main product of this reaction is a α-hydroxy ketone [3]. The reaction takes place in the presence of basic catalysts.

Acetol (hydroxyacetone) plays an important role as an intermediary in organic synthesis due to its ability to convert to a variety of products by dehydration, hydrogenation, oxidation, and polymerization [4]. Acrolein could be produced by its dehydration, while by its hydrogenation propylene glycol has been obtained. Among other compounds that can be obtained from its reactions are acetone, propionaldehyde, and furan derivates. High reactivity makes it valuable as an intermediary for esters, dioxane, α-amino ketones and α-amino alcohols. Furthermore, it is used in the food industry to give bread flavor and to induce flavor in milk compounds. In the cosmetic industry, it is used as a constituent of skin tanning agents.

Glycerol is one of the raw materials used to obtain the acetol in the presence of supported metals. Copper supported on acidic oxides such as Al_2_O_3_, ZrO_2_, Fe_2_O_3_, and SiO_2_ [5] is very selective towards acetol, while the strong acidic supports such as Keggin-type heteropoly acids and niobium oxides are more selective towards acrolein. The catalysts with high basicity are selective to allyl alcohol, as has been noticed by Kinage [6].

Spinel-type copper ferrites are an attractive class of materials, which are characterized by thermal stability, both redox and acid-base characteristics, and good catalytic properties for many reactions such as alcohol decomposition [7,8], selective oxidation [9,10,11], methanol steam reforming [12], water gas shift reaction [13], and ethanol oxidation [14]. Iron molybdates have been used as catalysts in photodegradation reactions [15].

The catalytic properties of these materials depend on cations distribution in the octahedral (Oh) and in the tetrahedral (Td) sites of the spinel lattice, respectively, on the presence of weak basic sites, and on a good dispersion of copper.

The purpose of this paper is to investigate the effects of molybdenum loading and of Fe/Cu ratio modification on the structural properties of copper ferrites and on the catalytic activity in the oxidative coupling of methanol with ethanol.

## 2. Results and Discussion

### 2.1. Catalysts Characterization

**Figure 1:**
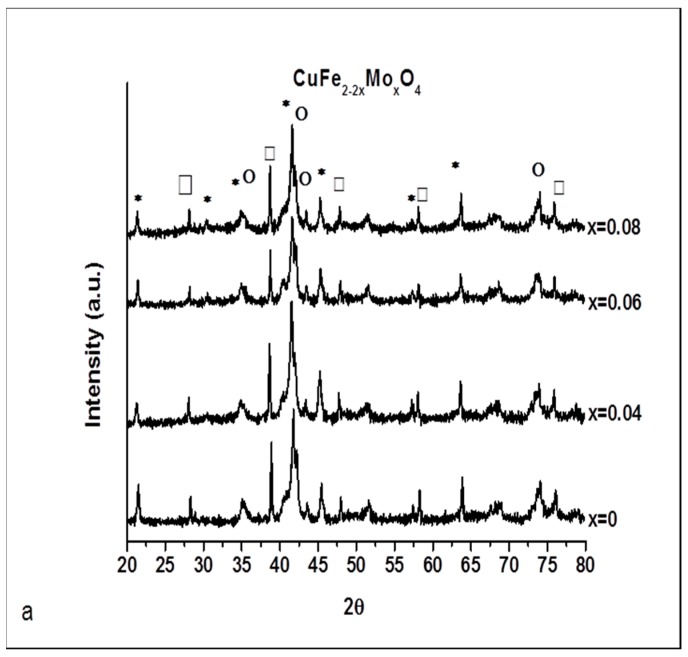
Patterns of the molybdenum doped copper ferrites: *–CuFe_2_O_4_, ○–Fe_3_O_4_ (magnetite), □–CuO (tenorite).

The PXRD (powder X-ray diffraction) (PANalytical, Almelo, The Netherlands) patterns of CuFe_2–2x_Mo_x_O_4_ samples are illustrated in Figure 1. From the diffraction patterns, lines corresponding to copper ferrite with spinel structure CuO (tenorite, PDF 00-041-0254) with a higher crystallinity F_3_O_4_ phase (magnetite, PDF 01-071-6336) superimposed with those corresponding to CuFe_2_O_4_ (PDF 00-034-0425) have been observed. The presence of diffraction lines corresponding to crystalline CuO and to small impurities of reduced Cu_2_O (2θ = 29, 34, 41, 62, 73°, JCPDS 05-0667) phase evidenced that not all Cu species are incorporated into the CuFe_2_O_4_ spinel. No lines corresponding to molybdenum have been highlighted, probably due to incorporation of Mo ions into the magnetite lattice. The peak positions are shifted to lower 2θ in the presence of molybdenum, which reveals the presence of microstrains in the structure such as lattice dislocation or oxygen vacancies [16]. 

The Brunauer–Emmett–Teller BET surface area for all samples evidenced that mixed oxides display smaller specific surface area, in the range 2.8–5.9 m^2^/g, that could be attributed to the presence of larger particles as well as to the existence of crystalline phases. 

**Figure 2:**
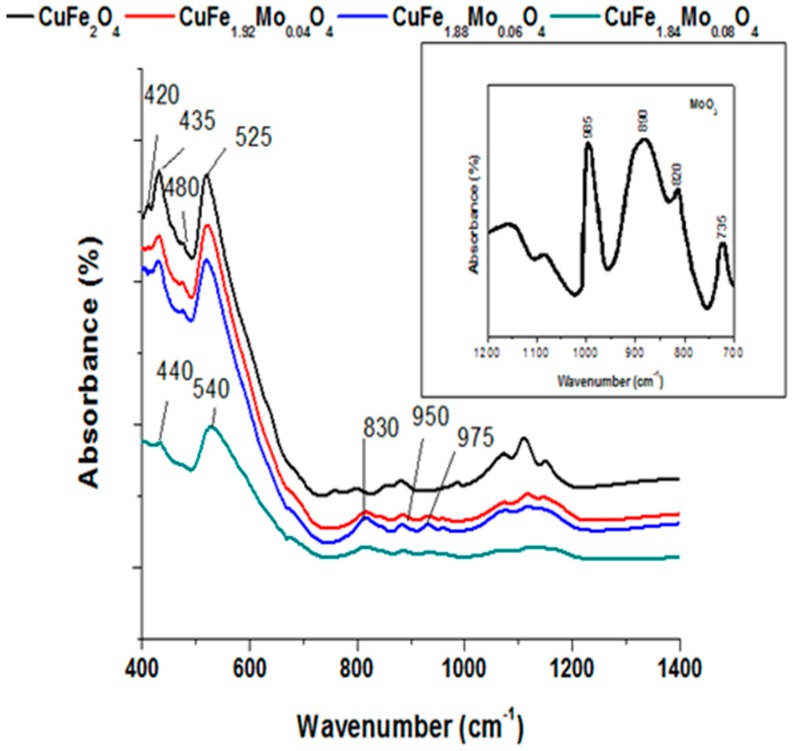
FT-IR spectra of the copper ferrites dopped with molybdenum.

FT-IR spectra (PerkinElmer, Fitchburg, USA) of samples are shown in Figure 2. The bands situated in the range 400–600 cm^–1^ are characteristic for Fe_3_O_4_, CuFe_2_O_4_, and CuO absorption. The band situated at 420 cm^–1^ corresponds to octahedral metal stretching of Cu^2+^–O^2–^, while Fe_2_O_3_ presents a vibration band at 480 cm^–1^ [17] and its presence is not observed on the sample with 0.08Mo. The strong absorption bands located at 525–540 cm^–1^ and those situated in the region 435–445 cm^–1^ are correlated with the presence of Fe–O bending vibration to magnetite and spinel copper ferrites [18] and are attributed to the tetrahedral and octahedral vibration of M^2+^/M^3+^ cations, respectively. The position of these bands is shifted to the higher wavenumber and their intensity decreases in the presence of molybdenum, probably due to the changes induced by the lattice modifications or by the defects in crystal like emergence of unsaturated sites on the surface. The molybdenum characteristic bands appear in the range 800–990 cm^–1^. The isolated tetrahedral molybdenum species MoO_4_^2–^ and two-dimensional polymeric forms to distorted octahedral molybdenum species have characteristic bands at 975 and 950 cm^–1^ [19]. The band located at 830 cm^–1^ is assigned to the antisymmetric mode of Mo–O–Mo in MoO_4_^2–^ (more pronounced for the sample with 0.06 Mo).

**Figure 3:**
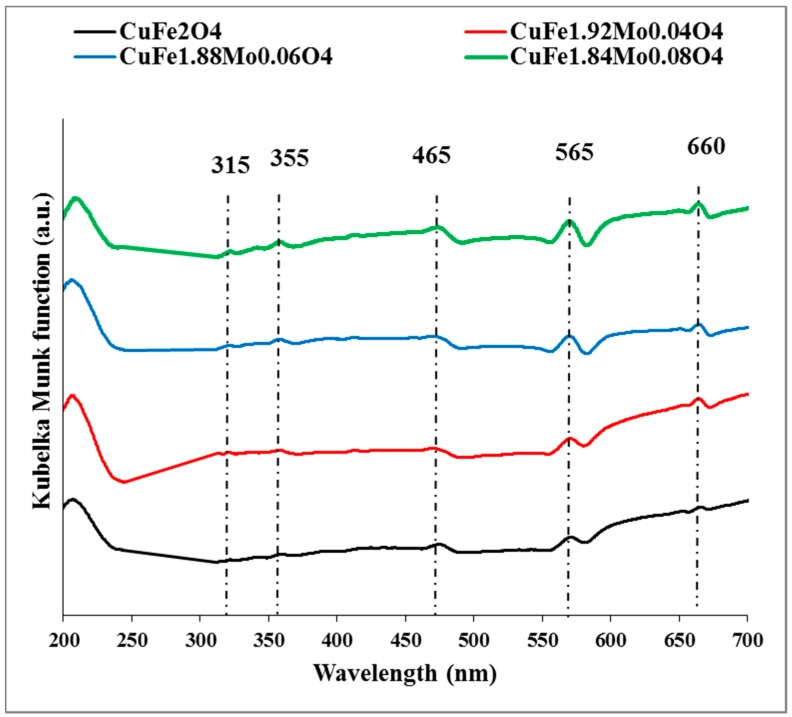
UV–Vis absorption spectra of the copper–molybdenum ferrites.

Figure 3 shows the UV–Vis absorption spectra (Shimadzu, Osaka, Japan) of copper–molybdenum ferrites. From the spectra, it was observed that the main absorption band of the samples appears in the wavelength region at 200–250 nm and corresponds to the small crystal size of the particles [20]. Absorption bands located at 300–360 nm have been assigned to an octahedral coordination of isolated molybdenum species [21]. The intensity of absorption band at 355 nm has been correlated with molybdenum loading on the surface. The band situated at 660 nm corresponds to d–d transition of Cu^2+^ ions in a distorted octahedral environment [22], but it could also be attributed to the interaction between the two metal, Fe–Cu. Iron oxides corresponding bands are located at 465 and 565 cm^−1^.

**Figure 4:**
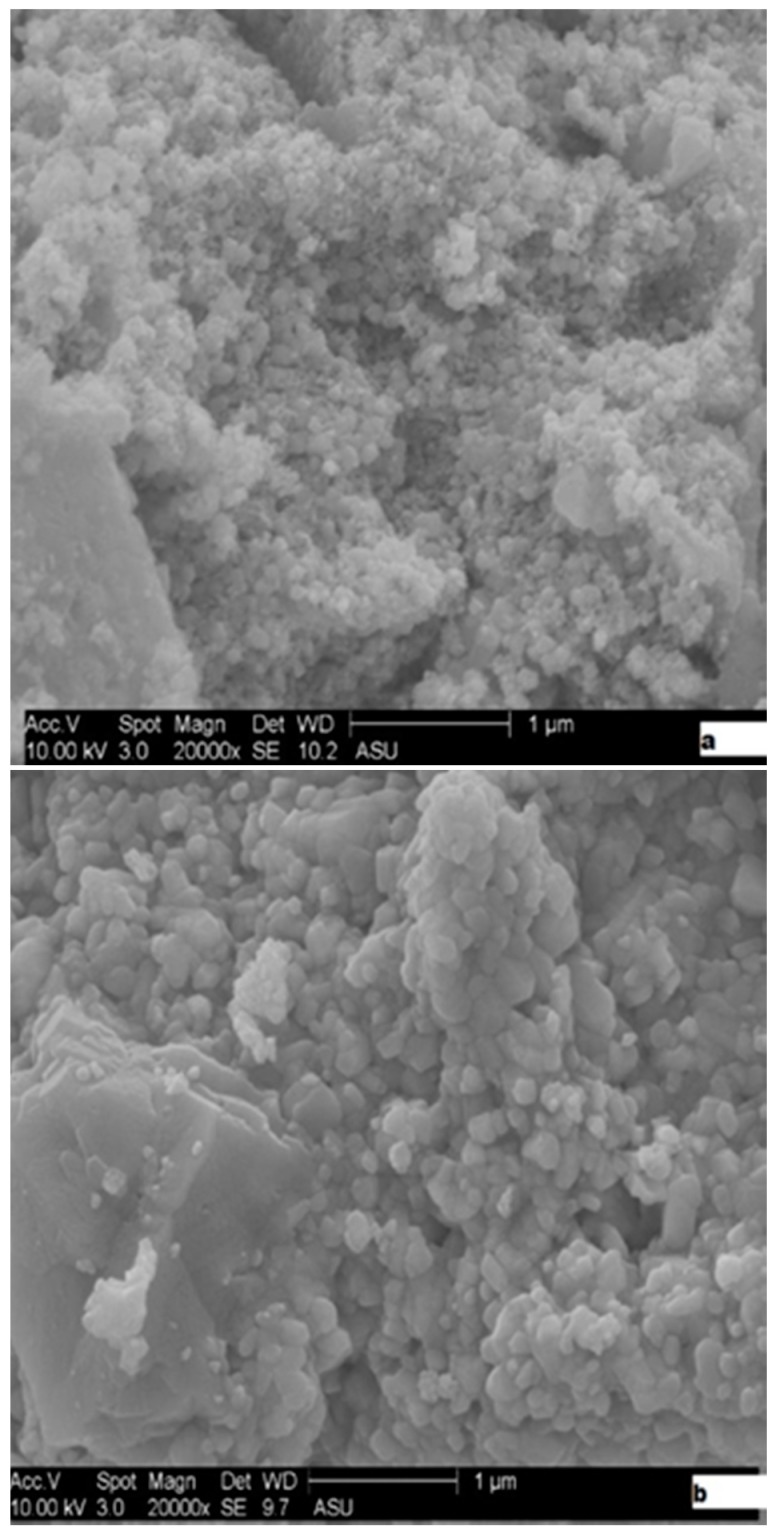

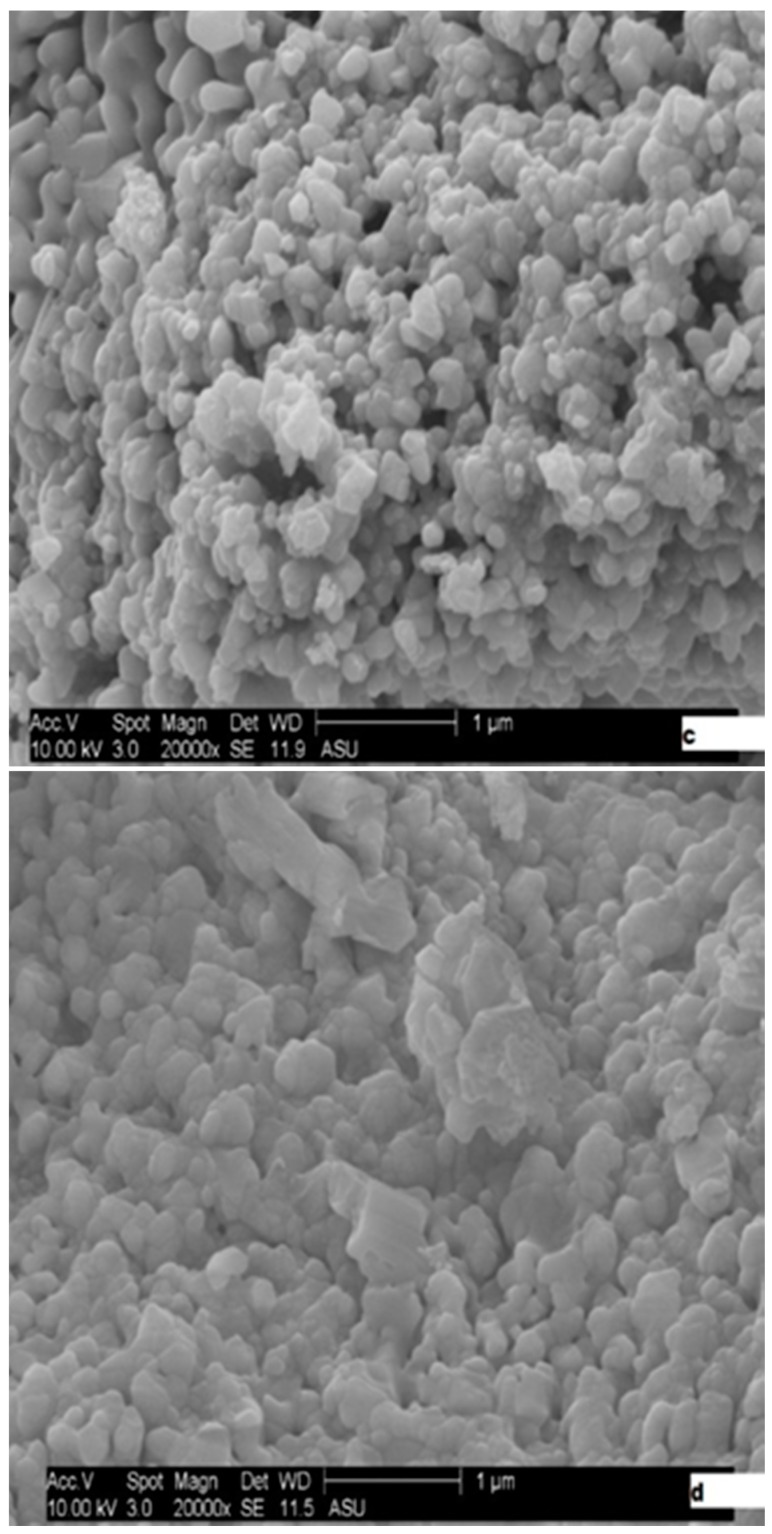

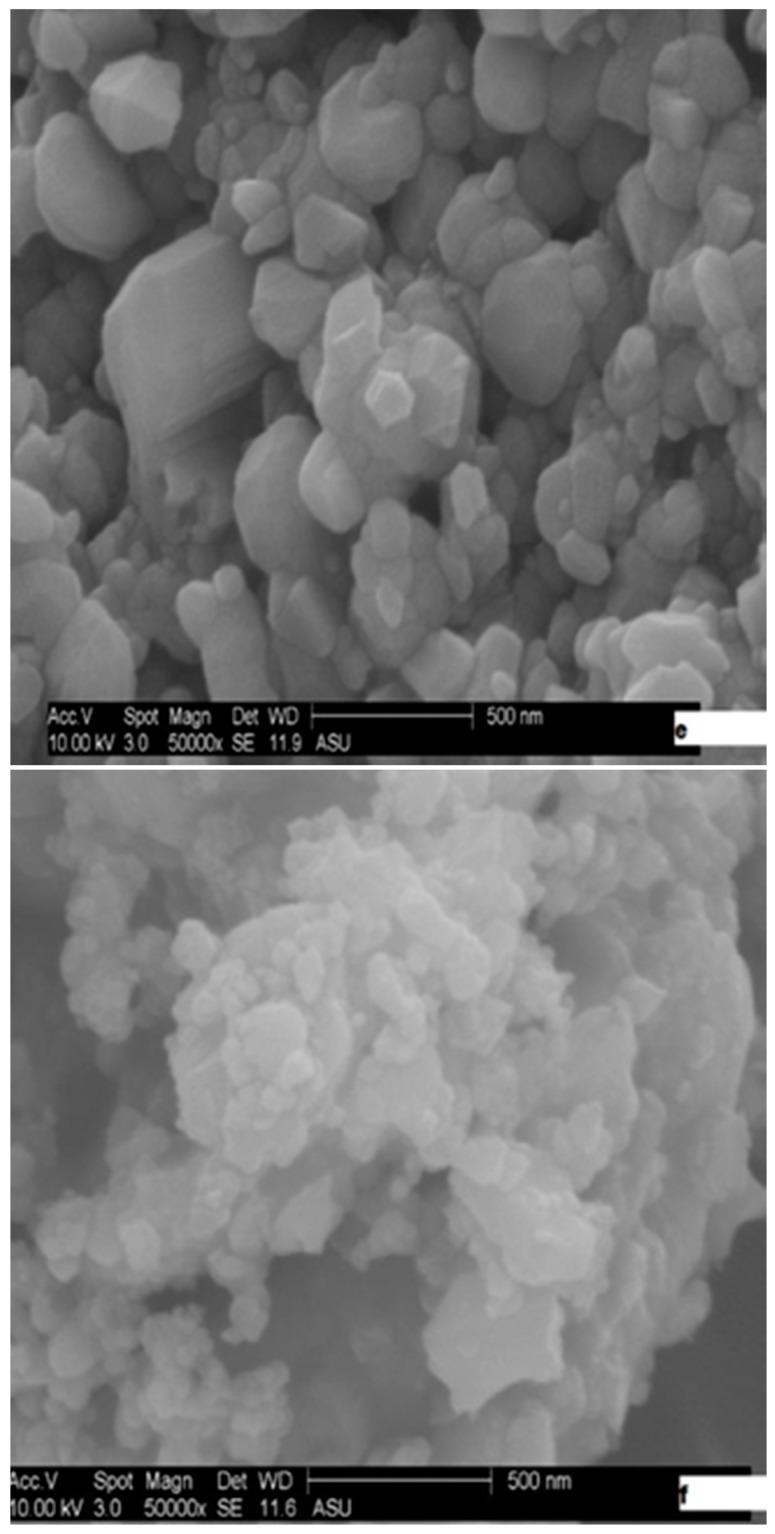
SEM images of the surface morphology of copper ferrites dopped with molybdenum (**a**) CuFe_2_O_4_; (**b**) CuFe_1.92_Mo_0.04_O_4_; (**c**) CuFe_1.88_Mo_0.06_O_4_; (**d**) CuFe_1.84_Mo_0.08_O_4_ 1 μm; (**e**) CuFe_1.88_Mo_0.06_O_4_; (**f**) CuFe_1.84_Mo_0.08_O_4_, 500 nm.

Scanning electron microscopy (SEM), (FEI Company, Hillsboro, OR, USA) images (Figure 4) highlight the presence of both disordered mesopores and macropores, remarking at the same time heterogeneous nucleation of particles with the appearance of spherical shape crystals. The sample without molybdenum shows the more uniform surface topography whereas in the presence of molybdenum grain agglomeration with an increase in the average particle size has been observed. The images were analyzed by Image J software in order to quantify the particle morphology and the average particle size was 41.3 nm (CuFe_2_O_4_), 60.9 nm (CuFe_1.92_Mo_0.04_O_4_), 73.1 nm (CuFe_1.88_Mo_0.06_O_4_), and 58.3 nm (CuFe_1.84_Mo_0.08_O_4_), respectively. 

Chemical composition of samples has been studied through Energy Dispersive Analysis spectrum (EDX) (FEI Company, Hillsboro, OR, USA) and the results are presented in Table 1. From these results the formulas for prepared samples are CuFe_1.93_O_4_, CuFe_1.2_Mo_0.03_O_1.98_, CuFe_1.35_Mo_0.06_O_1.66_, and CuFe_2.14_Mo_0.14_O_4.31_. The first sample has a formula very closed to the calculated one; the second and the third samples have values for Mo very close to the calculated ones and Fe and O values lower than those calculated; and the fourth sample has higher values than those calculated for Fe, Mo, and O.

**Figure 5:**
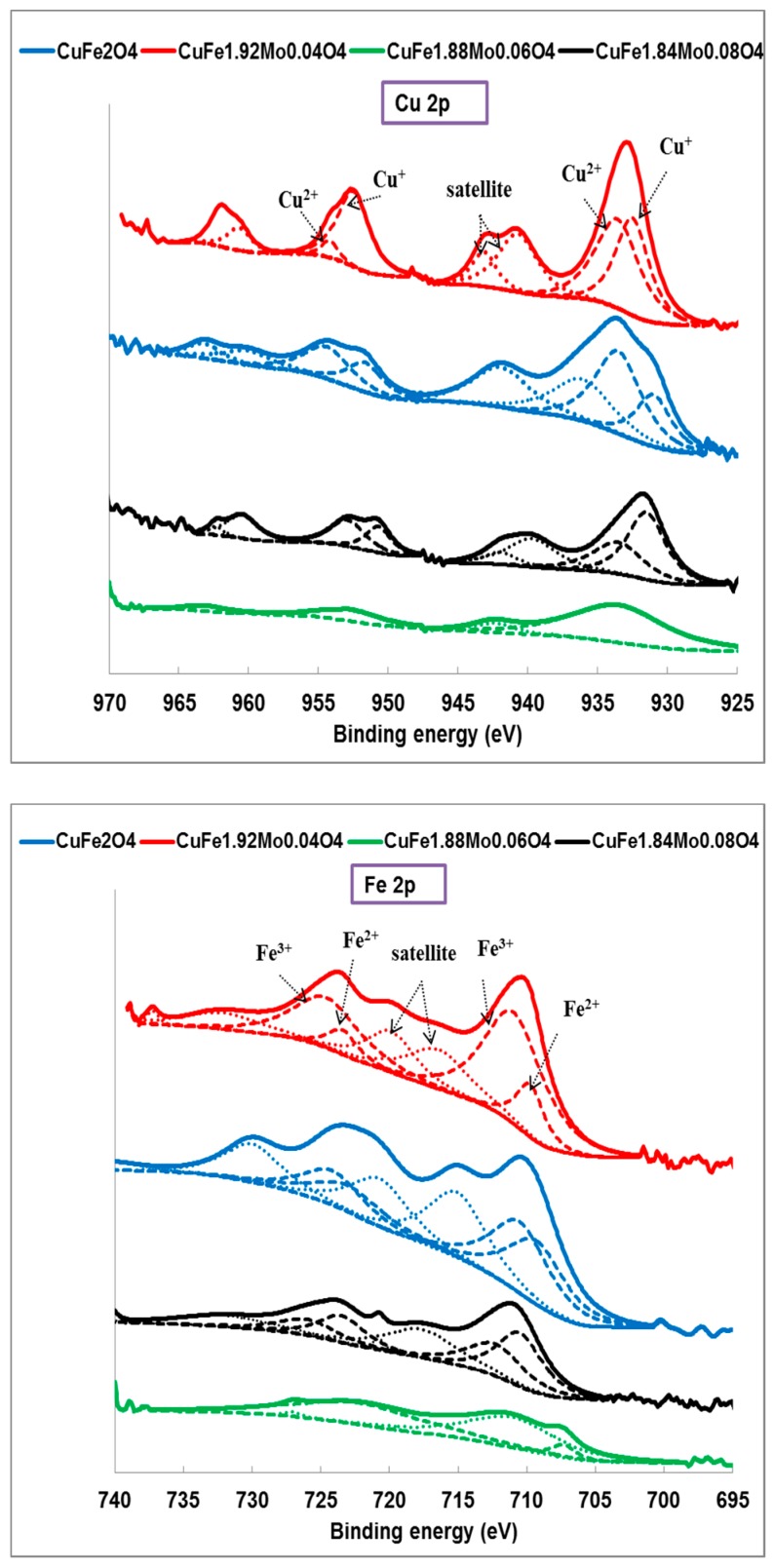

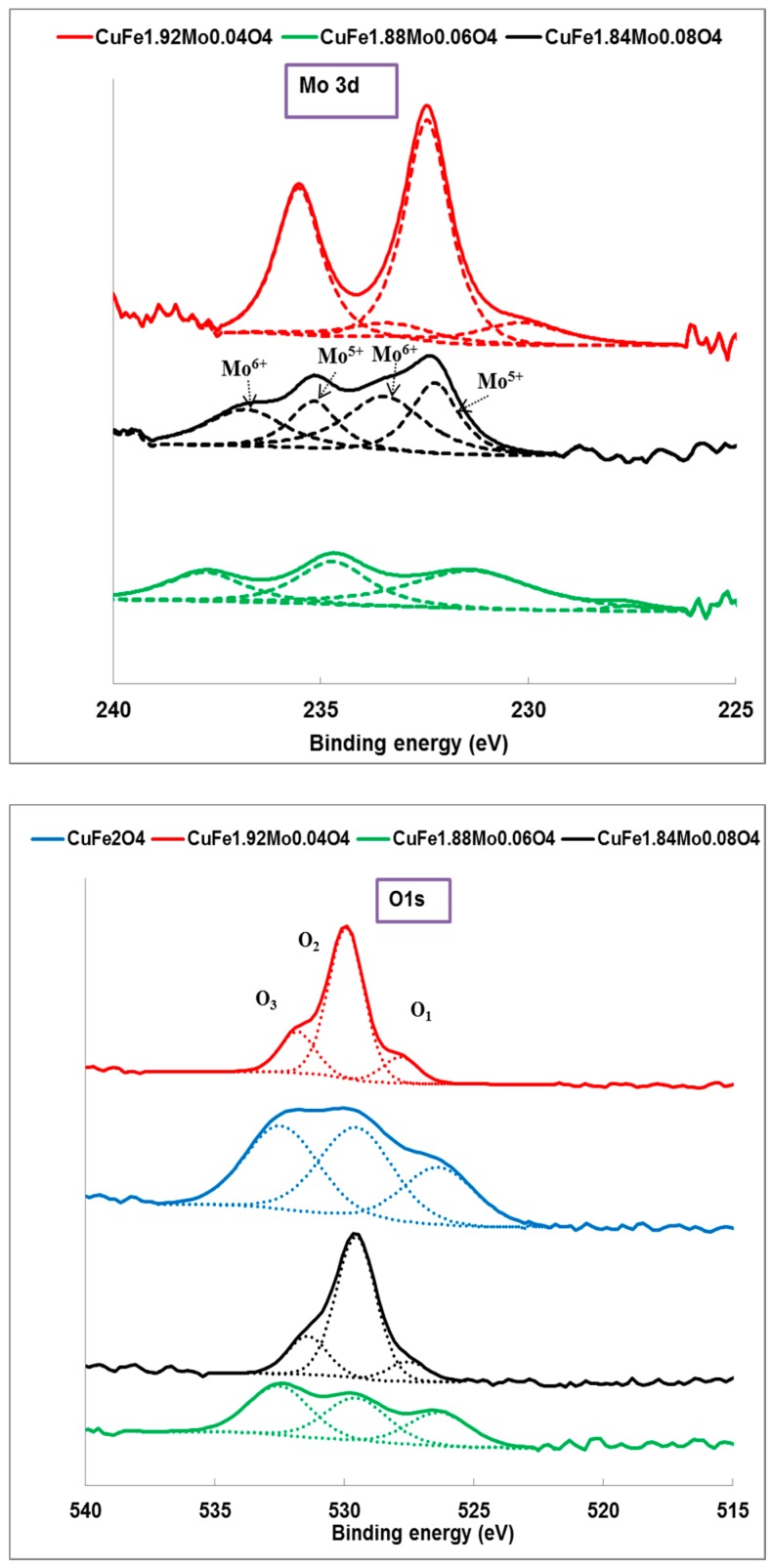
XPS spectra of copper ferrites dopped with molybdenum.

X-ray photoelectron spectroscopy (Kratos, Manchester, UK) results and surface composition estimated from XPS are shown in Figure 5 and Table 2. In order to calibrate the obtained spectra, the binding energies were corrected relative to the carbon 1 s signal at 284.8 eV. It could be observed that the binding energy positions of Cu2p are located at 930.9 eV (Cu 2p3/2) and 951.6 eV (Cu 2p1/2) corresponding to Cu^+^, the peaks at 933.5 eV (Cu 2p3/2) and 954.2 eV (Cu 2p1/2) are related to Cu^2+^ [23] and four satellites at 936, 941.9, 960.1, and 963.2 eV. The binding energies of Cu species indicate that in all samples both Cu^+^ and Cu^2+^ are present on the surface (except for the sample with 0.06Mo where only Cu^2+^ was present). The Fe 2p peaks are split into two components: Fe 2p3/2 and Fe 2p1/2. The peak assigned to metallic iron (Fe^0^) located at 707.3 eV and 722.2 eV is present on the sample with 0.06Mo (that contains Fe^0^ and Fe^3+^), while the other samples present peak at 709.8 (0.5) eV and 723.5 (± 0.7 eV) with two satellites corresponding to Fe^2+^ and the peaks associated to Fe^3+^ with a maximum at 710.5 (± 0.8) eV and 724.5 eV [24]. The Mo 3d spectrum is split into two components Mo 3d5/2 and Mo 3d3/2. The peaks of Mo^6+^ ion appear at 232.4 eV and 235.5 eV and are present in all samples with molybdenum. Mo^5+^ peaks are located at 230.2 and 233.3 eV and are present in the samples with 0.04 and 0.08Mo while in the sample with 0.06Mo appear peaks at 227.6 and 231.4 eV associated to Mo^4+^ [25]. The corresponding O1s spectra show three peaks: between 526.3–527.8 eV corresponding to lattice oxygen (O_latt1_) of copper oxides, 529.5–529.9 eV assigned to lattice oxygen (O_latt2_) of iron oxides, and at 531.4–532.4 eV, respectively, due to defect in oxide or to surface oxygen ions (O_ads_) [26,27]. As shown in Table 2, from the XPS spectrum of the last samples after catalytic reaction, an increase of Cu^2+^, Fe^3+^, and Mo^6+^, respectively, has been observed.

### 2.2. Catalysts Activity

**Figure 6:**
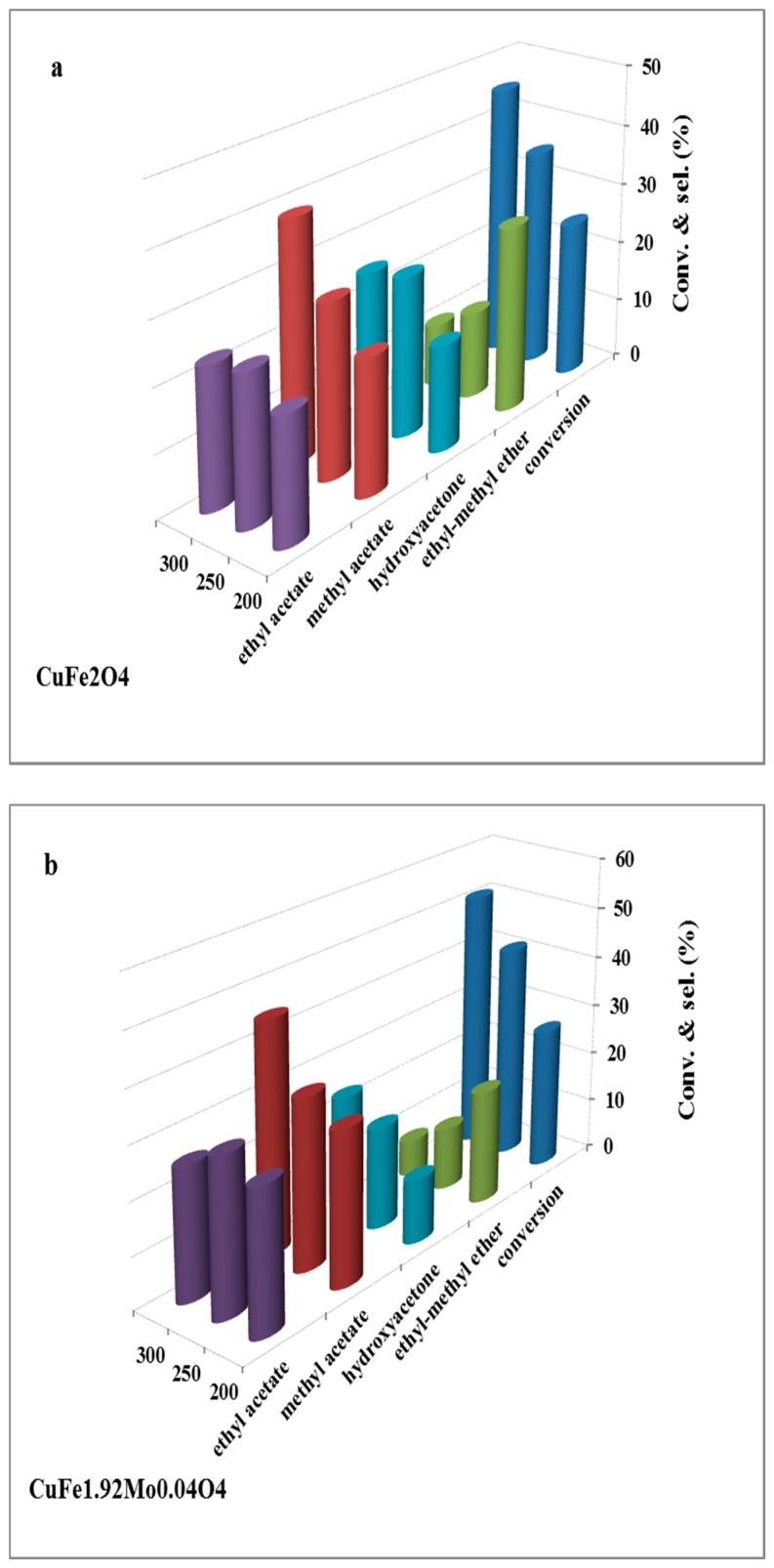

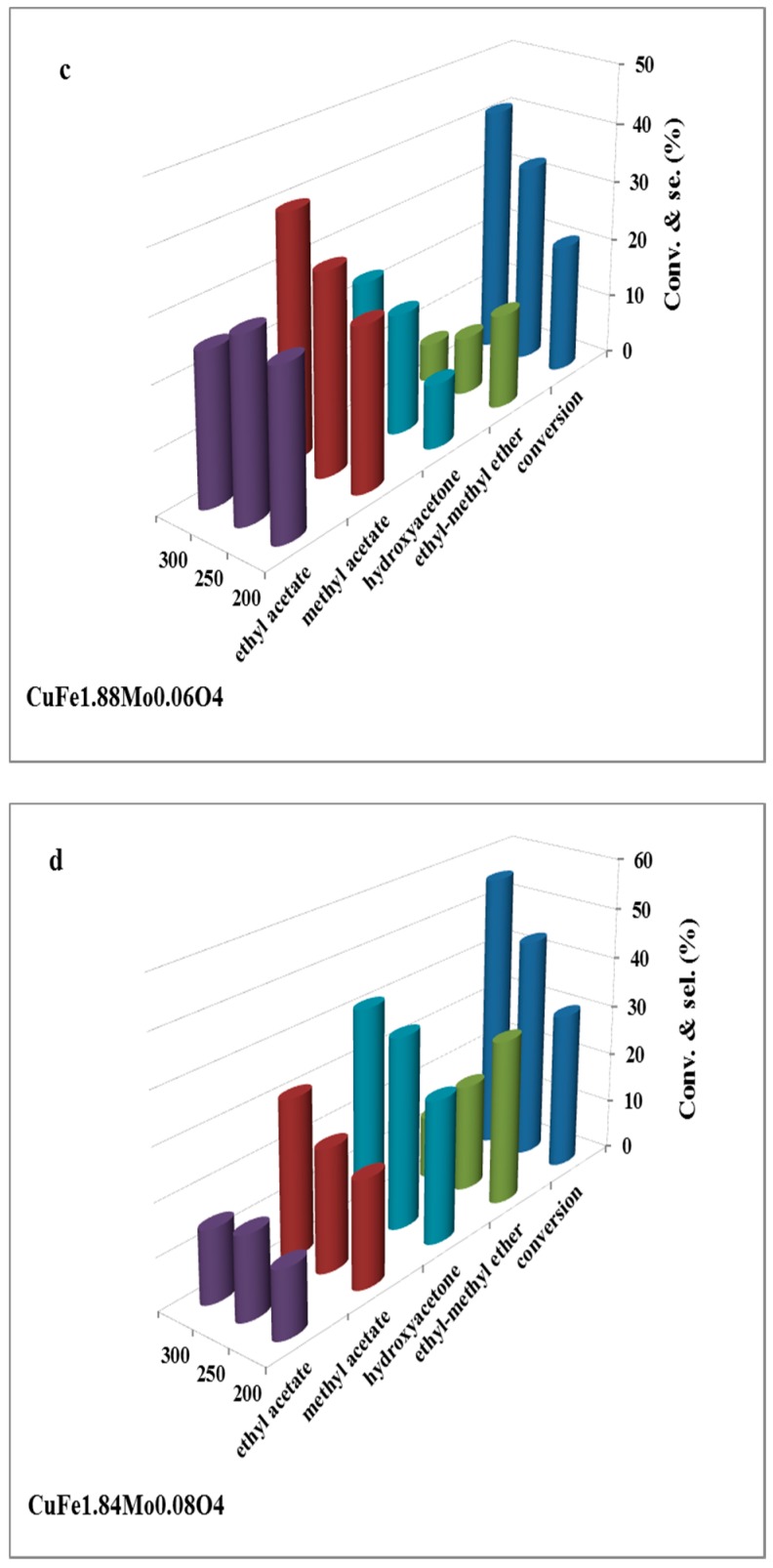

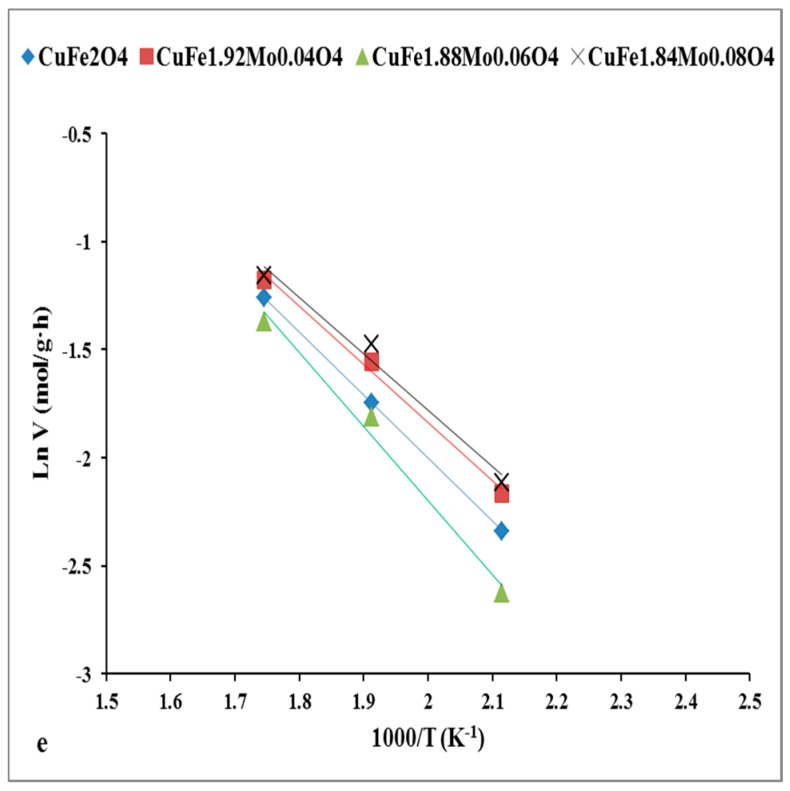
Catalytic performance of copper molybdenum ferrites in the oxidative coupling between methanol and ethanol as function of temperature (reaction conditions: molar ratio methanol:ethanol = 1; liquid fed 0.1 cm^3^/min; air flow 3000 cm^3^/min; catalyst amount 0.1 g), (**a**) CuFe_2_O_4_, (**b**) CuFe_1.92_Mo_0.04_O_4_, (**c**) CuFe_1.88_Mo_0.06_O_4_, (**d**) CuFe_1.84_Mo_0.08_O_4_, (**e**) Arrhenius plots for ethanol.

Initial catalytic experiments were carried out in order to investigate the influence of temperature on the catalytic activity and on the products’ selectivities. The main products for all catalytic reactions were hydroxyacetone (acetol), ethyl–methyl–ether, methyl acetate, ethyl acetate, and trace of diethyl–ether, dimethyl–ether, acetaldehyde, and formaldehyde. The ethanol conversion and the product distribution are illustrated in Figure 6. The conversion of ethanol has steadily increased with temperature. Concerning the behavior of the four catalysts, it has been observed that the ethanol conversion follows the order: CuFe_1.84_Mo_0.08_O_4_ > CuFe_1.92_Mo_0.04_O_4_ > CuFe_2_O_4_ > CuFe_1.88_Mo_0.06_O_4_. A good correlation between conversion of ethanol and molybdenum composition on the surface has been observed. The methyl–ethyl ether selectivity is higher at low temperature, dehydration reactions occurred faster than dehydrogenation in these conditions. Methyl acetate and hydroxyacetone selectivity increased with temperature, while ethyl acetate has the highest values at 250 °C. On the catalyst without molybdenum methyl–ethyl ether is the dominant product at low temperature, whilst at high temperature methyl acetate prevails. Esters are the predominant products on the samples with 0.04 and 0.06 Mo, and the sample with 0.08 Mo showed the active sites for hydroxyacetone and ether formation. 

The catalytic performances have been evaluated from the apparent activation energies, determined by the slopes of Arrhenius linear plots. The values of apparent activation energies are in the following order: 34, 32, 38, and 31 kJ/mol, respectively. 

**Figure 7:**
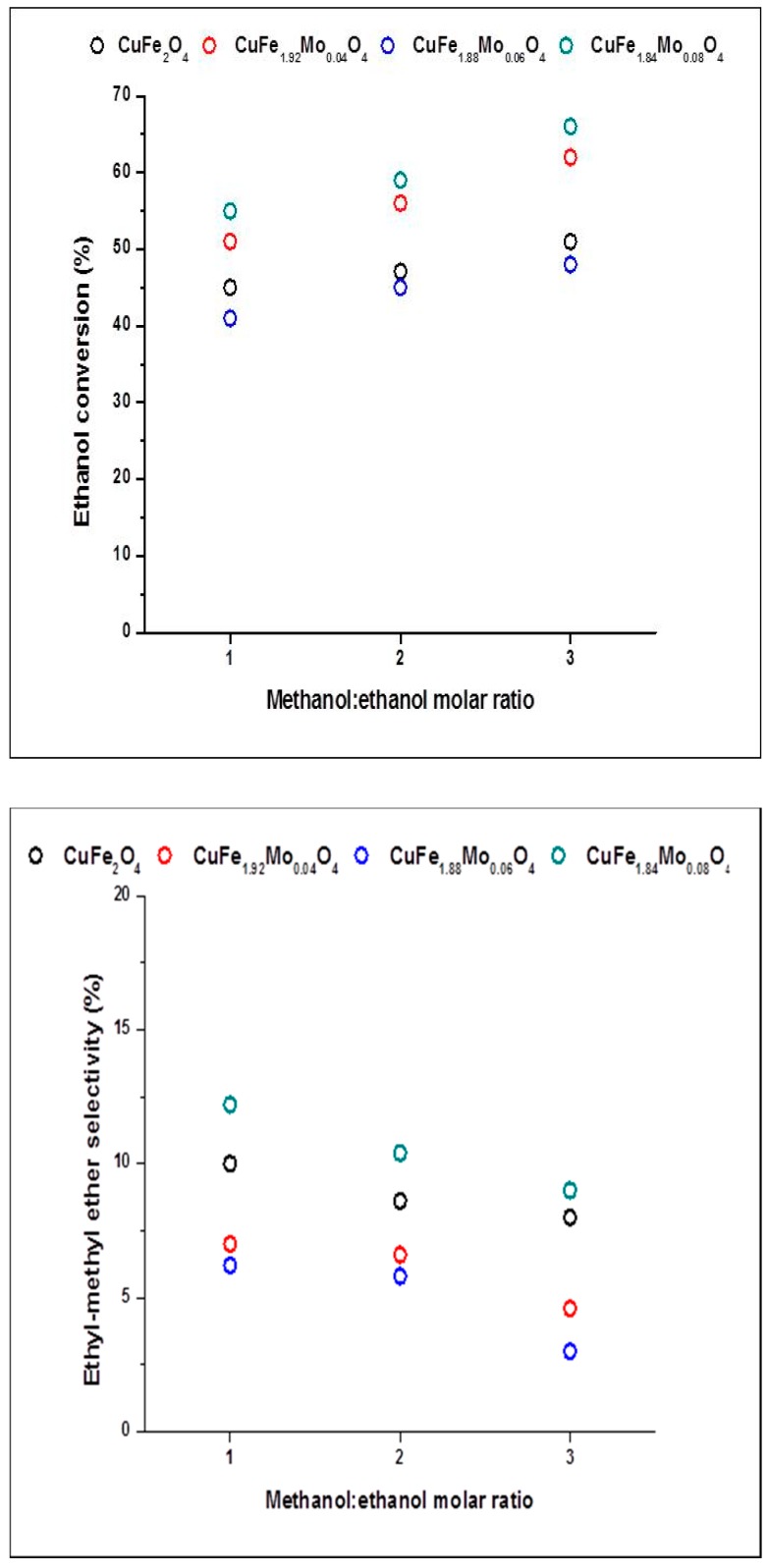

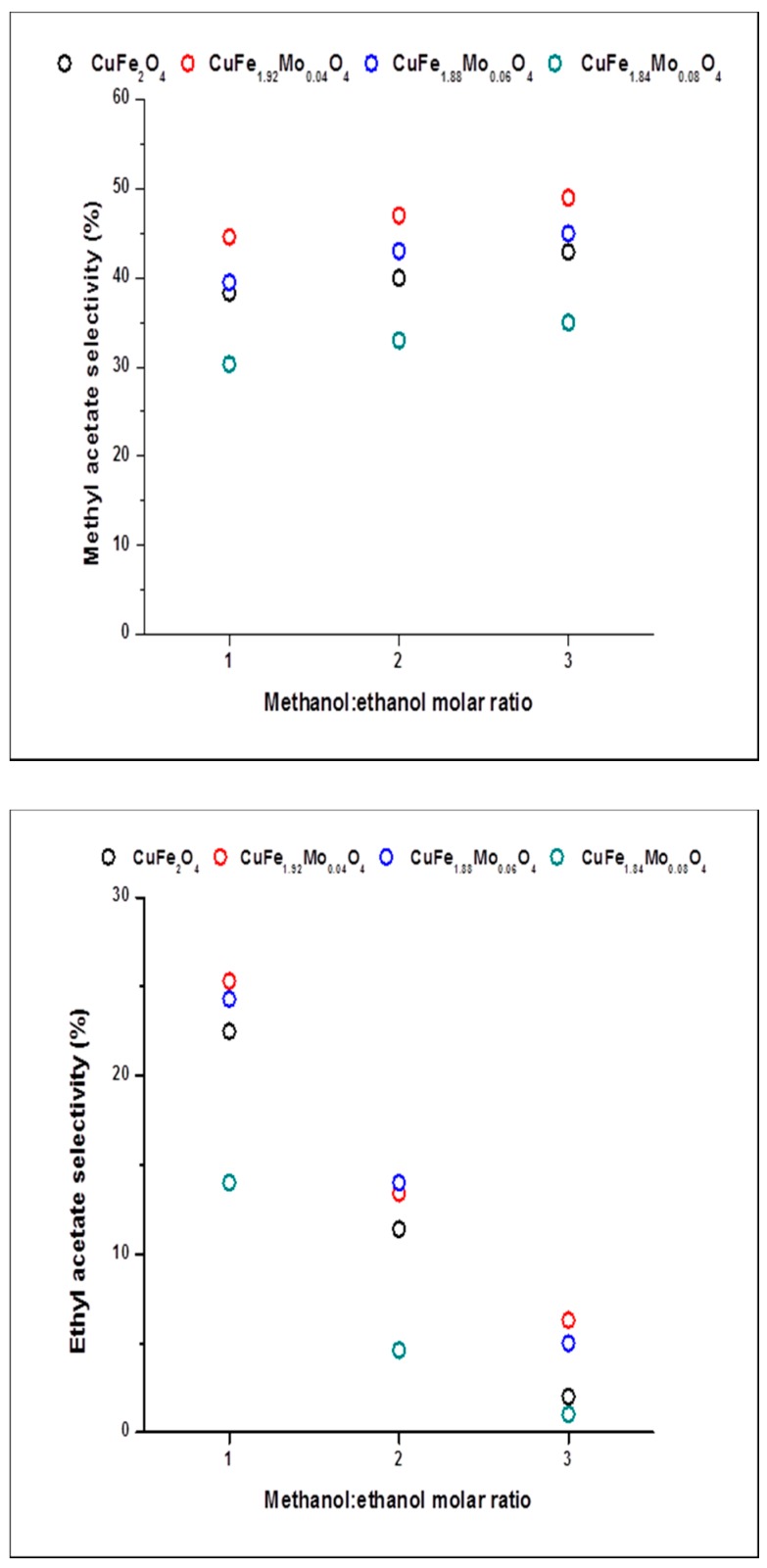

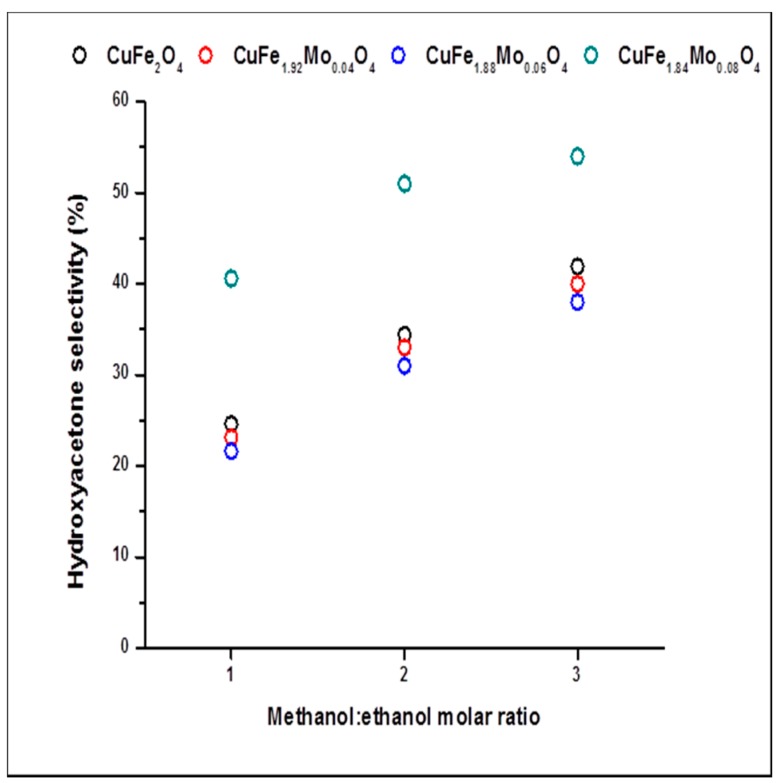
Catalytic performance of copper molybdenum ferrites in the oxidative coupling between methanol and ethanol as function of the methanol to ethanol molar ratio (reaction conditions: temperature 300 °C; liquid fed 0.1 cm^3^/min; air flow 3000 cm^3^/min; catalyst amount 0.1 g).(**a**) ethanol conversion, selectivity of (**b**) ethyl-methyl ether, (**c**) methyl-acetate, (**d**) ethyl-acetate, (**e**) hydroxyacetone.

The influence of methanol to ethanol molar ratio on the catalytic activity and on the products selectivities is shown in Figure 7. The presence of methanol in excess leads to an increase in both the catalytic activity and the selectivity to methyl acetate and hydroxyacetone, while the ether and ethyl acetate selectivities are decreasing.

### 2.3. Discussion

Our studies showed that the oxidative coupling and cross-acyloin condensation of methanol and ethanol in the gas phase to produce methyl or ethyl esters and hydroxyacetone are promoted effectively by the copper ferrites dopped with molybdenum. 

We noticed that there is a good correlation between catalytic activity and Cu^+^/Cu^2+^ Mo^5+^/Mo^6+^ ratio on the surface, respectively, the catalytic activity being directly proportional to these ratios. 

Similar results were obtained by Fan [28] in benzyl alcohol oxidation in the gas phase over mesoporous K–Cu–TiO_2_ catalysts with stable copper (I) oxidation state, noting that O_2_ acts as an efficient acceptor of hydrogen accelerating the reaction by creation of the free active Cu(I) sites.

The hydroxyacetone and ether selectivities are directly proportional to the Fe^2+^/Fe^3+^ ratio. However, the reaction perhaps does not occur only on these sites because the sample with 0.06Mo does not contain this kind of sites and probably on this catalyst the reaction takes place on Fe^0^ sites. 

Cu^2+^ and Fe^3+^ act as stronger Lewis acids compared with Cu^+^ and Fe^2+^, so for this reaction, the presence of weak Lewis acid sites is required. Reductions of Cu^2+^ to Cu^+^, Fe^3+^ to Fe^2+^, and Mo^6+^ to Mo^5+^ or Mo^4+^, respectively, take place by gaining an electron from interstitial oxygen. The loss of an electron leads to a high mobilization of the unstable interstitial oxygen that escapes from the lattice and becomes an active oxygen (O^–^).

Methanol and ethanol are converted in ethyl–methyl ether by dehydration or coupling reactions (1):

(1) $${\mathrm{CH}}_{3}\mathrm{OH}+{\;\mathrm{CH}}_{3}{\mathrm{CH}}_{2}\mathrm{OH}\;\to {\;\mathrm{CH}}_{3\;}{\mathrm{OCH}}_{2}{\mathrm{CH}}_{3}+{\;H}_{2}O\;$$

The ethyl–methyl ether formation takes place on oxides with acid–base properties and is favored by lower reaction temperatures. The ether formation involves the adsorption of two alcohol molecules on the neighbor active sites with different acid–base properties [29]. One alcohol molecule is adsorbed via the oxygen of the OH group on the weak Lewis acid sites and the second alcohol molecule is adsorbed on the strong basic site through the H of OH group with the formation of an alkoxide on the surface.

Acetaldehyde has been obtained from oxidation (or dehydrogenation) reaction and could react with a new molecule of methanol or ethanol via oxidative esterification giving methyl acetate and ethyl acetate (2), (3):

(2) $${\mathrm{CH}}_{3}\mathrm{CHO}+{\;\mathrm{CH}}_{3}\mathrm{OH}\;\to {\;\mathrm{CH}}_{3\;}{\mathrm{COOCH}}_{3}+{\;H}_{2}$$

(3) $${\mathrm{CH}}_{3}\mathrm{CHO}+{\;\mathrm{CH}}_{3}{\mathrm{CH}}_{2}\mathrm{OH}\;\to {\;\mathrm{CH}}_{3\;}{\mathrm{COOCH}}_{2}{\mathrm{CH}}_{3}+{\;H}_{2}$$

Methyl or ethyl formate has not been observed.

The aldehyde formation by dehydrogenation or by oxidation requires the presence of moderately basic sites (M^2+^–O^2–^). In the presence of aldehyde, the esters formation is accelerated with suppression of the secondary aldehydes’ oxidation to acids. The aldehyde adsorbed on the surface reacts with an alkoxy group with the formation of a hemiacetal which could eliminate another hydrogen to form an ester. Xu [30] has demonstrated that the reaction between the methoxy group and different aldehydes to form methyl esters takes place with good results on O/Au of nanoporous Au catalysts. Copper based catalysts are known to be active for both hydrogenation and dehydrogenation reactions, enhancing the C–C coupling reaction to esters [22]. 

The condensation of formaldehyde with acetaldehyde gives as a reaction product, by cross-acyloin condensation, hydroxyacetone (acetol) [3] (4):

(4) $${\mathrm{CH}}_{2}O+{\;\mathrm{CH}}_{3}\mathrm{CHO}\;\to {\;\mathrm{CH}}_{3\;}{\mathrm{COCH}}_{2}\mathrm{OH}\;$$

**Scheme 1:**
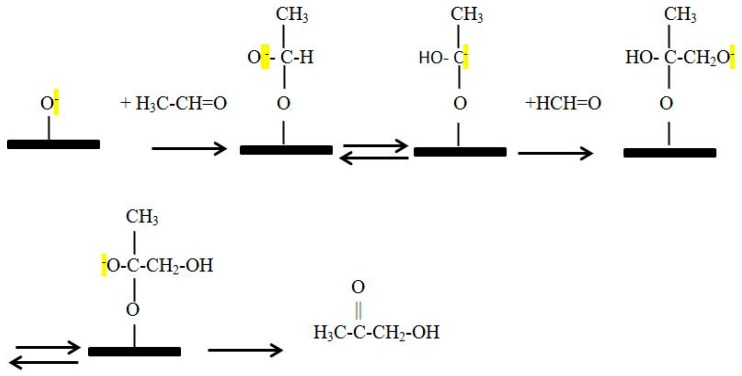
The reaction mechanism of acyloin condensation of acetaldehyde with formaldehyde.

In Scheme 1 we propose a mechanism for acyloin condensation of formaldehyde with acetaldehyde to hydroxyacetone.

Acetaldehyde is adsorbed on the surface through oxygen leading to the formation of an oxoanion which can accept a proton from carbon, making it a carbanion. The formed carbanion reacts with formaldehyde with the new C–C bond formation and finally to hydroxyacetone.

## 3. Experimental Part

### 3.1. Materials and Catalysts’ Synthesis

For the catalysts’ synthesis and for catalytic reaction, the following chemicals were used: copper nitrate Cu(NO_3_)_2_·3H2O (CAS No.10031-43-3, Carl Roth), ammonium molybdate, (NH_4_)_6_Mo_7_O_24_·4H_2_O (CAS No: 12054-85-2, FlukaChemika), iron citrate tribasic monohydrate, C_6_H_5_FeO_7_·H_2_O (CAS No. 3522-50-7, Sigma-Aldrich), urea (CAS No. 57-13-6, Chimopar), methanol p.a. 99.3% (CAS No.67-56-1, Consors), and ethanol p.a. 99.5% (CAS No. 64-17-5, SC Chimreactiv).

Copper ferrites dopped with molybdenum, with the formula CuFe_2–2x_Mo_x_O_4_ (x = 0, 0.04, 0.06 and 0.08), were prepared by substitution of Fe^3+^ ions with Mo^6+^. To this end, a mixed aqueous solution of copper nitrate, iron citrate, and ammonium molybdate was used as reagent over which an aqueous solution of urea was added as an organic fuel. The resulting solution by mixing all components was heated at 80–85 °C until the water was evaporated. The viscous gel obtained was dried at 100 °C for 24 h and then was calcined at 200 °C, 400 °C, and 600 °C for 2 h at each temperature.

### 3.2. Catalysts’ Characterization

The Brunauer–Emmet–Teller (BET) surface area and pore size distribution were measured by a Micromeritics ASAP 2020 Surface area and Porosity Analyzer, at 77 K. Before the sorption measurements samples were degassed under vacuum at 200 °C for 8 h.

The X-ray diffraction patterns were obtained with a PANalyticalX’Pert Pro MRD diffractometer with a Cu K_α_ radiation. Samples were scanned in the range 5–80° with a step size of 0.0251°.

The particle size and the morphology of samples were characterized by SEM with an XL-30 Environmental SEM using 20KeV on samples ground and prepared to a standard.

Infrared spectra were obtained with a Bruker IFS 66 V/S vacuum spectrometer with a diamond attenuated total reflectance (ATR) and with a MIR DLaTGS detector.

The UV-vis-NIR spectra were determined with a UV3600 UV-vis spectrophotometer with a Shimadzu ISR-3100 integrating sphere attachment; the angle of incident light was of 0–8°; the wavelength range was 200–700 nm; two light sources were employed: A D_2_ (deuterium) lamp for the ultraviolet range and a WI (halogen) lamp for the visible and near-infrared range. The samples, before the analysis, were diluted with extra pure barium sulfate (purchased from NacalaiTesque).

X-ray photoelectron spectroscopic (XPS) measurements were carried out using a VG-220IXL spectrometer with a monochromated Al Kα radiation (1486.6 eV, line width 0.8 eV). The pressure in the analyzing chamber was kept at the level of 10–9 torr while recording the spectra. The spectrometer has the energy resolution of 0.4 eV. All the binding energies were corrected with reference to C(1s) at 284.8 eV. Deconvolution of the spectrum was done using the CASAXPS software with the accuracy of 0.2 eV. Shirley background was used for the deconvolution.

### 3.3. Catalytic Experiments

The activity measurements for oxidative methanol–ethanol coupling were carried out in a flow reactor equipped with electronic control of reaction temperature at atmospheric pressure. Reaction temperature was varied in the range 200–300 °C; the catalyst weight was of 0.1 g. Liquid mixture of two alcohols (methanol and ethanol) with different molar ratios (methanol: ethanol ratio was varied in the range 1–3) was fed to the reactor with a pump. The air flow was 3000 cm^3^/h and the liquid mixture was fed with 0.1 cm^3^/ min. The reaction products were collected periodically and analyzed by gas chromatography with a GC K072320 Thermo-Quest chromatograph equipped with a FID detector. The main products were hydroxyacetone, methyl acetate, ethyl acetate, and ethyl–methyl ether. Acetaldehyde and formaldehyde were also detected in much lower concentrations.

## 4. Conclusions

Copper ferrites dopped with molybdenum are found to be active catalysts for the gas phase oxidative coupling between methanol and ethanol. The main phases that have been highlighted by XRD are copper ferrite with spinel structure, CuO (tenorite) very well crystallized, and magnetite. Molybdenum is present as isolated tetrahedral species MoO_4_^2–^ at a lower Mo content and polymeric octahedral species correlated with an increase in molybdenum content. 

The catalytic properties are influenced by the acid–base properties of ferrites, by the composition of defects in oxide, and by the surface oxygen ions. The catalyst with higher Cu^+^/Cu^2+^ and Mo^5+^/Mo^6+^ ratios is more active, while the catalyst with higher Fe^2+^/Fe^3+^ ratio is more selective to hydroxyacetone. The presence of Cu^+^, Fe^2+^, and Mo^5+^ or Mo^4+^ that act as weak Lewis acid sites is required to lead to a high mobilization of the interstitial oxygen that becomes an active oxygen. This study helped us to understand the oxidative coupling reaction over copper molybdenum ferrites.

## Figures and Tables

**Table 1:** Chemical composition of copper ferrites substituted with molybdenum.

Catalyst	Wt (%)				At (%)			
	Cu	Fe	Mo	O	Cu	Fe	Mo	O
CuFe_2_O_4_	27.1	45.3	–	26.9	14.6	27.7	–	57.6
CuFe_1.92_Mo_0.04_O_4_	38.7	40.5	1.7	19.1	24.0	28.5	0.7	46.8
CuFe_1.88_Mo_0.06_O_4_	37.2	43.7	3.6	15.4	24.7	33.0	1.6	40.7
CuFe_1.84_Mo_0.08_O_4_	24.0	45.0	5.2	25.9	13.3	28.2	1.9	56.7

**Table 2:** Surface composition estimated from XPS.

Catalyst	Cu^+^/Cu^2+^	Fe^2+^/Fe^3+^	Mo^5+^(Mo^4+^)/Mo^6+^	Surface Composition At (%)		
				Cu	Fe	Mo
CuFe_2_O_4_	0.5	0.9	–	9.1	18.2	0
CuFe_1.92_Mo_0.04_O_4_	0.8	0.2	0.2	9.7	12.2	4.6
CuFe_1.88_Mo_0.06_O_4_	0	0	(0.05)	8.7	13.2	4.3
CuFe_1.84_Mo_0.08_O_4_	1.6	1.3	0.8	8.4	14.5	4.8
CuFe_1.84_Mo_0.08_O_4_ (used)	1.3	1.2	0.7	8.5	14.5	4.9
